# Gait asymmetry as a determinant of functional recovery and return to sport after ACL reconstruction: a cross-sectional biomechanical analysis

**DOI:** 10.3389/fbioe.2026.1762965

**Published:** 2026-05-13

**Authors:** Ali Ibrahim Alhefzi, Ravi Shankar Reddy, Fareed F. Alfaya, Shaker Hassan S. Alshehri, Debjani Mukherjee, Batool A. Alkhamis, Irshad Ahmad, Saleh Kardm, Ghada Mohamed Koura, Zuhair Al Salim, Ajay Prashad Gautam, Faisal M. Alyazedi, Sara Mohamed Samir, Ahmed Mohamed Fathi Elshiwi, Ahmed M. El Melhat

**Affiliations:** 1 Department of Orthopedic Surgery, College of Medicine, King Khalid University, Abha, Saudi Arabia; 2 Program of Physical Therapy, Department of Medical Rehabilitation Sciences, College of Applied Medical Sciences, King Khalid University, Abha, Saudi Arabia; 3 Department of Surgery, College of Medicine, Najran University, Najran, Saudi Arabia; 4 Department of Sport Science and Physical Activity, University of Hafr Al Batin, Hafar Al-Batin, Saudi Arabia; 5 Department of Physical Therapy, Prince Sultan Military College of Health Sciences, Dhahran, Saudi Arabia; 6 Department of Physical Therapy for Musculoskeletal Disorders and its Surgery, Faculty of Physical Therapy, Cairo University, Cairo, Egypt; 7 Department of Medical Rehabilitation, College of Applied Medical Sciences, Najran University, Najran, Saudi Arabia; 8 Department of Physical Therapy, Saudi German Hospital, Aseer, Saudi Arabia; 9 Department of Physical Therapy, Faculty of Health Sciences, Beirut Arab University, Beirut, Lebanon

**Keywords:** anterior cruciate ligament reconstruction, biomechanical Phenomena, gait analysis, rehabilitation, return to sport

## Abstract

**Introduction:**

Gait asymmetry and impaired functional performance are common after anterior cruciate ligament (ACL) reconstruction and may influence return-to-sport (RTS) outcomes. Despite advancements in rehabilitation, a substantial proportion of individuals fail to regain pre-injury activity levels, often due to unresolved biomechanical deficits. Quantifying gait asymmetry and its relationship with functional capacity may provide insight into factors that differentiate those who successfully return to sport from those who do not.

**Methods:**

Eighty-six participants (43 RTS, 43 non-RTS) between 6 and 24 months post-ACL reconstruction completed 3D gait analysis and a standardized battery of single-leg hop tests. Gait asymmetry indices included kinetic and kinematic variables, while functional performance was assessed using hop distances, timed hops, and limb symmetry indices (LSIs). Group comparisons were analyzed using independent *t*-tests, and relationships were examined using Pearson correlations and multiple linear regression.

**Results:**

The RTS group demonstrated significantly lower asymmetry across all gait variables, including vertical ground reaction force asymmetry expressed as an asymmetry index percentage derived from body-weight–normalized force values (8.45% ± 3.21% vs. 12.34% ± 4.02%, p = 0.001) and peak knee flexion angle asymmetry (degrees; 3.12 ± 1.10° vs. 5.67 ± 1.45°, p < 0.001). They also performed better on all hop tests, including single-leg hop distance (centimeters; 162.34 ± 12.45 vs. 148.76 ± 14.33 cm, p < 0.001) and Limb Symmetry Index for the 6-m timed hop (percentage ratio of involved-to-uninvolved limb performance; 96.45% ± 2.85% vs. 88.34% ± 3.65%, p < 0.001). Gait asymmetry metrics showed moderate-to-strong correlations with hop performance (r = −0.58 to 0.44). Regression analysis identified peak knee flexion angle asymmetry (B = −2.45, β = −0.41, p < 0.001) and knee extensor moment asymmetry (B = −8.12, β = −0.39, p = 0.004) as significant predictors.

**Conclusion:**

Gait asymmetry differentiates individuals based on RTS status and significantly predicts functional performance after ACL reconstruction. These findings support the clinical integration of gait analysis to guide rehabilitation and RTS decisions.

## 1 Introduction

Anterior cruciate ligament (ACL) injuries are among the most prevalent and functionally limiting musculoskeletal injuries in young and physically active populations, particularly athletes (Ezzat et al., 2021). Surgical reconstruction followed by progressive rehabilitation is widely accepted as the standard approach to restoring joint stability and enabling a return to pre-injury levels of activity (Jenkins et al., 2022). However, despite advances in surgical technique and postoperative care, a substantial proportion of individuals do not successfully return to sport (RTS) at their prior level of participation (Paterno et al., 2022). This failure is often not due to structural failure of the graft but rather to unresolved deficits in neuromuscular control, movement biomechanics, and lower-limb function (Costa et al., 2022). Among these factors, gait asymmetry—defined as the uneven distribution of biomechanical loads or movements between limbs during walking or running—has emerged as a critical marker of incomplete recovery that may influence long-term outcomes, including functional performance, reinjury risk, and quality of life (Knobel et al., 2021).

Persistent gait asymmetries have been shown to remain even after individuals meet standard clinical milestones following ACL reconstruction (Bri et al., 2022; Chen et al., 2025). Prior studies have demonstrated that inter-limb differences in joint loading and energy absorption strategies are associated with altered movement mechanics and reduced functional capacity, which may ultimately affect RTS outcomes (Bri et al., 2022; Chen et al., 2025). In parallel, research on hop test performance has shown that asymmetrical limb loading is associated with deficits in strength, power, and neuromuscular efficiency, reinforcing the clinical relevance of symmetry-based assessments, such as limb symmetry indices and gait parameters, in evaluating RTS readiness (Buckthorpe et al., 2024; Maestroni and Read, 2025). Despite growing evidence supporting the importance of both gait biomechanics and functional performance, existing studies have predominantly examined these domains in isolation (Bertozzi et al., 2023; Roy et al., 2025). As a result, it remains unclear whether specific gait asymmetry parameters independently predict functional outcomes or differentiate RTS status. This limitation is particularly evident in the lack of integrated analyses combining three-dimensional gait asymmetry metrics with standardized hop performance measures. A more precise understanding of the relationship between gait asymmetry and functional performance is needed, especially across different stages of post-ACL reconstruction recovery (Buckthorpe et al., 2024).

While prior research has established the individual relevance of gait asymmetry and hop performance, their combined role in distinguishing RTS outcomes remains insufficiently explored (Fjellman-Wiklund et al., 2022). In particular, limited evidence exists linking specific biomechanical variables—such as ground reaction force, knee flexion angle, and stance time—to functional measures including hop distance and symmetry (Garcia et al., 2022). Clarifying these associations may support clinical decision-making by identifying objective biomechanical markers of recovery and functional readiness (Hughes and Dai, 2023). Accordingly, the present study aimed to (i) compare gait asymmetry and hop performance between individuals who returned to sport and those who did not following ACL reconstruction, and (ii) examine the relationship between specific gait asymmetry variables and functional hop performance in individuals 6–24 months post-surgery.

## 2 Materials and methods

### 2.1 Study design, ethics, and settings

This cross-sectional study was conducted between 5 December 2023, and 10 August 2024, at the Sports Injury Rehabilitation Clinic, Department of Physical Therapy and Health Rehabilitation Sciences, College of Applied Medical Sciences, King Khalid University, Kingdom of Saudi Arabia. Ethical approval was obtained from the Institutional Review Board of King Khalid University (Reference No. ECM#2023-3209), and written informed consent was obtained from all participants prior to data collection. The study adhered to the ethical principles outlined in the Declaration of Helsinki.

### 2.2 Sample size calculation

*A priori* power analysis was conducted using G*Power (version 3.1.9.7) for a two-tailed independent t-test comparing RTS and non-RTS groups. Assuming a moderate effect size (d = 0.60), an alpha level of 0.05, a power of 0.80, and a 1:1 allocation ratio, the required sample size was 72 participants. To account for potential data loss, the final sample included 86 participants.

### 2.3 Participants

Participants were recruited through consecutive sampling from the patient registry and clinician referrals at the Sports Injury Rehabilitation Clinic at King Khalid University between December 2024 and April 2025. All individuals were screened based on a confirmed diagnosis of unilateral ACL rupture treated surgically with primary ACL reconstruction using either hamstring or patellar tendon grafts. Diagnosis was confirmed by an orthopedic specialist through clinical examination and supported by magnetic resonance imaging (MRI) findings (Shantanu et al., 2021). Eligible participants were between 18 and 35 years of age, physically active before injury, and at 6–24 months post-surgery at the time of assessment. Inclusion criteria required participants to have completed a full postoperative rehabilitation program, be free of any graft failure or re-injury, and demonstrate sufficient pain-free range of motion to perform functional tests. Participants were also required to be medically cleared for functional testing by their treating physician.

Exclusion criteria included a history of bilateral knee injuries, concomitant grade III injury to other knee ligaments, symptomatic meniscal tears requiring surgical intervention, or any neurological or musculoskeletal conditions affecting lower-limb function unrelated to ACL injury. Additionally, individuals who had undergone revision ACL reconstruction or presented with significant gait abnormalities unrelated to ACL pathology were excluded. All eligible participants underwent a standardized pre-assessment screening that included medical history review, physical examination, and verification of rehabilitation completion. Only participants who met all eligibility criteria and provided informed consent were enrolled in the study and scheduled for gait and functional performance testing. All participants completed their postoperative rehabilitation at the same Sports Injury Rehabilitation Clinic following institutionally standardized, criterion-based ACL rehabilitation protocols. Although individual progression rates and session attendance may have varied, rehabilitation phases, therapeutic exercise frameworks, and return-to-activity criteria were guided by consistent clinical protocols supervised by licensed physical therapists. Medication use and session frequency were not prospectively controlled due to the cross-sectional design. However, time since surgery, graft type, range of motion, and isokinetic strength were recorded to account for clinically relevant variables.

### 2.4 Gait asymmetry indices

Gait asymmetry was evaluated using three-dimensional motion analysis during level overground walking at a self-selected speed (Sedaghati et al., 2025). Participants ambulated along a straight 10-m walkway, which ensured enough distance for gait initiation and termination outside the main capture zone, maintaining steady-state walking during data collection. The motion capture system and embedded force plates were positioned in the central section of the walkway to record consistent mid-gait cycles, thereby reducing the effects of acceleration and deceleration. Data were gathered using a three-dimensional motion capture system with infrared cameras (Simi Motion Systems Ltd., Unterschleißheim, Germany) (Schweizer et al., 2022), synchronized with embedded force plates (Kistler Instrument AG, Winterthur, Switzerland). The integrated system facilitated the concurrent collection of kinematic data from reflective markers and kinetic data from ground reaction forces via inverse dynamics. Synchronization and integration of kinetic–kinematic data were performed in accordance with validated laboratory protocols for combined motion capture and force plate analysis. Reflective markers were placed on standardized anatomical landmarks according to the Plug-in Gait full-body model.

Participants wore standardized athletic footwear to control for variability in foot-ground interaction. Gait asymmetry indices included vertical ground reaction force (GRF) asymmetry, peak knee flexion angle asymmetry, knee extensor moment asymmetry, stance time asymmetry, step length asymmetry, knee valgus angle asymmetry, hip–knee–ankle alignment asymmetry, and pelvic drop asymmetry. Vertical ground reaction force values were first normalized to body weight (%BW) before calculating the asymmetry index to enable comparability across participants. Knee extensor moments were normalized to body mass (Nm/kg) prior to computing the percentage asymmetry index. Asymmetry was determined as the absolute percentage difference between limbs using the formula: Asymmetry Index (%) = |Involved − Uninvolved|/((Involved + Uninvolved)/2) × 100. All gait variables reported in the Results and Table 2 are presented as asymmetry index values in percentages. The units in parentheses (e.g., degrees, centimeters, Nm/kg, %) indicate the original measurement units of the underlying variables. The reported values reflect percentage inter-limb asymmetry rather than raw differences. Higher values denote greater inter-limb disparity. Data were averaged across trials, with at least five valid gait cycles analyzed per participant to ensure reliability (Schweizer et al., 2022). All valid steady-state gait cycles with complete kinematic and kinetic data were included in the analysis. A minimum of five clear gait cycles per limb was required for inclusion; if more than five valid cycles were recorded, all acceptable cycles were retained.

For each participant, the outcome variables were averaged across included gait cycles to generate a single representative value per limb, which was then used to calculate asymmetry indices. Vertical ground reaction force asymmetry was derived from peak vertical force values measured with synchronized force plates and expressed as a percentage of the inter-limb difference (Schweizer et al., 2022). Knee extensor moments were calculated using inverse-dynamics procedures embedded in the motion-analysis software and normalized to body mass (Nm/kg) to account for inter-individual anthropometric differences (Schweizer et al., 2022). Kinematic variables, including peak knee flexion angle, knee valgus angle, hip–knee–ankle alignment, and pelvic drop, were measured in degrees from three-dimensional marker trajectories (Schweizer et al., 2022). Spatiotemporal variables such as stance time and step length were derived from gait cycle events detected via force plate contact timing and marker displacement data, with stance time asymmetry expressed as a percentage and step length asymmetry in centimeters (Schweizer et al., 2022).

### 2.5 Functional performance (hop tests)

Functional performance was assessed using a standardized battery of single-limb hop tests, including the single-leg hop for distance, triple hop for distance, crossover hop for distance, and the 6-m timed hop (Sedaghati et al., 2025). During the crossover hop test, participants performed three consecutive forward hops on the same limb while alternately crossing over a straight line marked on the floor. Each landing had to be stabilized for at least 2 s to be considered valid. The cumulative horizontal distance covered across the three hops was measured in centimeters. All hop tests were conducted in accordance with established ACL rehabilitation guidelines to ensure standardized task execution and scoring procedures (Sedaghati et al., 2025; Schweizer et al., 2022). Testing was performed on a flat, non-slip laboratory-grade surface to ensure consistent landing conditions and reduce external variability. Each participant completed three trials per limb for all hop tests. For the distance-based tests (single-leg, triple, and crossover hops), the longest successfully completed distance (cm) was recorded as the best performance. For the 6-m timed hop, the fastest successfully completed time (seconds) was recorded. Only the single best trial per limb was retained for statistical analysis, consistent with established ACL functional testing recommendations (Berg et al., 2022). Distances were measured using a calibrated floor tape, and time was recorded using a digital stopwatch. Standardized verbal instructions and demonstrations were provided, and trials were repeated if balance was not maintained upon landing.

### 2.6 Limb symmetry index (LSI)

For each hop test, a Limb Symmetry Index (LSI) was calculated to quantify inter-limb functional symmetry (Simonsson et al., 2025). The LSI was defined as: LSI (%) = (Involved Limb Performance/Uninvolved Limb Performance) × 100 (Kumar Sr et al., 2026), and values were expressed as percentages. Although an LSI of ≥90% is commonly cited in clinical practice as a benchmark for satisfactory symmetry during return-to-sport evaluation (Simonsson et al., 2025), LSIs in the present study were analyzed as continuous variables rather than dichotomized at a fixed threshold. No inclusion or exclusion decisions were based on a specific cut-off value, thereby preserving statistical sensitivity and avoiding over-reliance on arbitrary categorization. LSIs were calculated for each of the four hop tests to provide a comprehensive assessment of functional recovery. Distance-based hop outcomes were measured in centimeters using calibrated floor markings, and the 6-m timed hop was recorded in seconds using a digital stopwatch. For the timed hop, the involved-to-uninvolved limb ratio was maintained to ensure that higher LSI percentages consistently reflected better functional symmetry despite the inverse relationship between time and performance.

### 2.7 Return-to-sport status

RTS classification was determined using a structured interview that quantified pre-injury sport participation and post-operative activity levels. Pre-injury participation was defined as engagement in organized sport or structured physical activity at a minimum frequency of two sessions per week for at least six consecutive months prior to injury (Andrew et al., 2012). Participants were classified as RTS if they had resumed the same sport at the same competitive or recreational level and were participating at ≥90% of their pre-injury training frequency (sessions per week), including unrestricted involvement in training and/or competition (Andrew et al., 2012). Participants were classified as non-RTS if they had not returned to their primary sport, had discontinued structured sport participation, or had resumed participation at <90% of their pre-injury training frequency or at a lower competitive level. This classification approach ensured objective alignment between pre-injury exposure and post-reconstruction return-to-participation status. Participants were categorized as RTS if they had resumed unrestricted involvement in training or competition in their primary sport at the same level as before injury. This classification method is consistent with the criteria used in prior outcome-based ACL research (Rodriguez-Merchan and Valentino, 2022). The non-RTS group included participants who had not returned to any competitive or recreational sport or had returned to it at a reduced level of participation.

### 2.8 Demographic and clinical characteristics

Demographic and clinical data were collected at baseline. They included age, sex, body mass index (BMI), time since surgery (months), involved limb, graft type (hamstring or patellar tendon), and pre-injury sport level (recreational or competitive). Pre-injury sport level was determined using a structured self-report questionnaire administered during baseline assessment. Participants were classified as Competitive if they were engaged in organized sport with regular team or individual competition and structured training ≥4 sessions per week prior to injury. Participants were classified as Recreational if they participated in physical activity or sport ≤3 sessions per week without formal competitive involvement. This classification approach was adopted to distinguish structured, performance-based athletic participation from general fitness or leisure activity. Isokinetic quadriceps and hamstring strength were measured using an isokinetic dynamometer (Biodex System 4) at 60°/s in a seated position, and results were normalized to body weight and reported in Nm/kg. Isokinetic dynamometry testing was performed prior to the functional hop assessments to minimize the potential influence of fatigue from dynamic performance tasks on strength measurements. Standardized warm-up procedures were completed before testing, and adequate rest intervals were provided between strength and functional assessments. Passive range of motion (ROM) for knee flexion and extension was assessed using a standard universal goniometer, with participants positioned according to established clinical measurement procedures to ensure consistent anatomical alignment and identification of the joint axis (Norkin and White, 2016).

### 2.9 Data analysis

All statistical analyses were conducted using IBM SPSS Statistics version 24.0 (IBM Corp., Armonk, NY, USA). Before analysis, the data were screened for normality using the Shapiro–Wilk test and visual inspection of histograms, confirming that all continuous variables were normally distributed, thus permitting the use of parametric statistical methods. Descriptive statistics were calculated for all variables and presented as means and standard deviations for continuous data, and as frequencies and percentages for categorical data. Independent-samples t-tests were used to compare gait asymmetry indices and functional performance outcomes between participants who returned to sport and those who did not. Categorical variables, such as sex, graft type, and sport level, were analyzed using chi-square tests to assess group differences. To evaluate associations between gait asymmetry variables and hop test performance, Pearson correlation coefficients were calculated. Variables showing significant bivariate correlations were further examined using multiple linear regression analyses to identify significant predictors of functional performance, with unstandardized coefficients (B), 95% confidence intervals, and standardized beta values (β) reported. Statistical significance for all analyses was set at p < 0.05.

## 3 Results

### 3.1 Demographic and clinical characteristics

Participant characteristics were well balanced between the return-to-sport (RTS) and non-return-to-sport (non-RTS) groups, with no significant differences observed in age (25.42 ± 3.21 vs. 26.14 ± 3.78 years, p = 0.384), sex distribution (26/17 vs. 24/19 male/female, p = 0.834), or BMI (23.87 ± 2.45 vs. 24.12 ± 2.67 kg/m^2^, p = 0.543). Time since surgery was similar across groups (13.52 ± 4.89 vs. 14.01 ± 5.34 months, p = 0.621), as were graft type, involved limb, pre-injury sport level, and isokinetic strength values (all p > 0.29), indicating that the groups were demographically and clinically comparable (Table 1). An exploratory within-group comparison of gait asymmetry and hop performance between male and female participants was conducted descriptively. Although small numerical differences were observed in selected asymmetry parameters, no consistent sex-specific pattern emerged across gait or functional outcomes within either the RTS or non-RTS groups. Due to the limited subgroup sample sizes and the absence of an *a priori* power calculation for sex-based comparisons, these findings should be interpreted with caution and were not included in the primary inferential analyses.

**TABLE 1:** Demographic and clinical characteristics of participants (RTS vs. non-RTS).

Variable	RTS (n = 43)	Non-RTS (n = 43)	p-value
Age (years)	25.42 ± 3.21	26.14 ± 3.78	0.384
Sex (male/female)	26/17	24/19	0.834
BMI (kg/m^2^)	23.87 ± 2.45	24.12 ± 2.67	0.543
Time since surgery (months)	13.52 ± 4.89	14.01 ± 5.34	0.621
Involved limb (right/Left)	22/21	21/22	0.841
Graft type (hamstring/patellar)	30/13	29/14	0.792
Pre-injury sport level (recreational/competitive)	18/25	20/23	0.642
Isokinetic quadriceps strength (Nm/kg)	2.45 ± 0.31	2.41 ± 0.35	0.472
Isokinetic hamstring strength (Nm/kg)	1.82 ± 0.28	1.78 ± 0.25	0.398
Knee flexion ROM (degrees)	135.72 ± 4.15	134.89 ± 4.64	0.364
Knee extension ROM (degrees)	0.56 ± 1.12	0.74 ± 1.25	0.298

RTS, return to sport; BMI, body mass index; ROM, range of motion; Nm/kg; N m per kilogram.

### 3.2 Gait asymmetry comparison

Gait asymmetry was significantly higher in the non-RTS group across all measured variables. Notable differences included vertical ground reaction force asymmetry (12.34% ± 4.02% vs. 8.45% ± 3.21%, mean difference = −3.89, 95% CI: −5.74 to −2.04, p = 0.001) and peak knee flexion angle asymmetry (5.67 ± 1.45° vs. 3.12 ± 1.10°, mean difference = −2.55, 95% CI: −3.21 to −1.89, p < 0.001). Additional variables such as step length asymmetry (4.12 ± 1.33 vs. 2.56 ± 1.14 cm, p < 0.001) and pelvic drop asymmetry (2.15 ± 0.71 vs. 1.23 ± 0.64°, p < 0.001) also showed significant group differences, indicating that reduced gait asymmetry is linked to a successful return to sport (Table 2). When analyses were stratified by pre-injury sport level (Recreational vs. Competitive), the direction and extent of between-group differences in gait asymmetry (RTS vs. non-RTS) remained consistent across both subgroups. Competitive participants exhibited numerically lower asymmetry values in the RTS group than in the non-RTS group across the primary variables (vertical ground reaction force, peak knee flexion angle, and knee extensor moment asymmetry), and similar patterns were observed within the Recreational subgroup. However, due to smaller sample sizes after stratification, these analyses were deemed exploratory and were not sufficiently powered for definitive subgroup conclusions.

**TABLE 2:** Comparison of gait asymmetry indices between RTS and non-RTS groups.

Variable	RTS (n = 43)	Non-RTS (n = 43)	Mean difference	95% CI	p-value
Vertical ground reaction force asymmetry (%)	8.45 ± 3.21	12.34 ± 4.02	−3.89	(-5.74, −2.04)	0.001
Peak knee flexion angle asymmetry (°)	3.12 ± 1.10	5.67 ± 1.45	−2.55	(-3.21, −1.89)	<0.001
Knee extensor moment asymmetry (Nm/kg)	0.28 ± 0.08	0.34 ± 0.09	−0.06	(-0.10, −0.02)	0.004
Stance time asymmetry (%)	6.78 ± 2.13	9.43 ± 2.56	−2.65	(-3.89, −1.41)	0.002
Step length asymmetry (cm)	2.56 ± 1.14	4.12 ± 1.33	−1.56	(-2.17, −0.95)	<0.001
Knee valgus angle asymmetry (°)	2.13 ± 0.95	3.01 ± 1.02	−0.88	(-1.44, −0.32)	0.006
Hip-knee-ankle alignment asymmetry (°)	1.85 ± 0.76	2.67 ± 0.89	−0.82	(-1.35, −0.29)	0.003
Pelvic drop asymmetry (°)	1.23 ± 0.64	2.15 ± 0.71	−0.92	(-1.27, −0.57)	<0.001

RTS, return to sport; Nm/kg, N m per kilogram; cm, centimeter; °, degrees; %, percent; CI, confidence interval.

All variables are presented as asymmetry index percentages calculated using the formula described in Section 2.4. Units shown in parentheses indicate the original measurement unit of the underlying biomechanical variable prior to asymmetry index computation.

### 3.3 Functional performance (hop tests)

**FIGURE 1:**
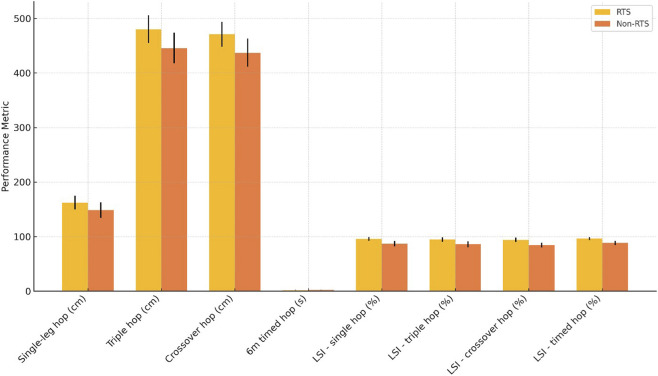
Comparison of functional hop performance between RTS and non-RTS groups, including distances, timed hops, and limb symmetry indices.

The RTS group showed significantly better hop performance across all tests. The single-leg hop distance was greater in the RTS group (162.34 ± 12.45 vs. 148.76 ± 14.33 cm, mean difference = 13.58, 95% CI: 7.94 to 19.22, p < 0.001), and similar patterns appeared for the triple hop (480.21 ± 25.32 vs. 445.67 ± 28.21 cm, p < 0.001) and crossover hop (470.78 ± 22.67 vs. 437.12 ± 25.87 cm, p < 0.001). The 6-m timed hop was completed more quickly by RTS participants (1.92 ± 0.15 vs. 2.13 ± 0.18 s, mean difference = −0.21, 95% CI: −0.29 to −0.13, p < 0.001). All limb symmetry indices were notably higher in the RTS group, ranging from 93.89% to 96.45%, compared to 84.45%–88.34% in the non-RTS group (Table 3; Figure 1).

**TABLE 3:** Comparison of functional performance (hop tests) between RTS and non-RTS groups.

Variable	RTS (n = 43)	Non-RTS (n = 43)	Mean difference	95% CI	p-value
Single-leg hop for distance (cm)	162.34 ± 12.45	148.76 ± 14.33	13.58	(7.94, 19.22)	<0.001
Triple hop for distance (cm)	480.21 ± 25.32	445.67 ± 28.21	34.54	(22.10, 46.98)	<0.001
Crossover hop for distance (cm)	470.78 ± 22.67	437.12 ± 25.87	33.66	(21.78, 45.54)	<0.001
6-M timed hop (seconds)	1.92 ± 0.15	2.13 ± 0.18	−0.21	(-0.29, −0.13)	<0.001
Limb symmetry index - single hop (%)	95.78 ± 3.45	87.12 ± 4.78	8.66	(6.74, 10.58)	<0.001
Limb symmetry index - triple hop (%)	94.56 ± 4.10	85.89 ± 5.45	8.67	(6.55, 10.79)	<0.001
Limb symmetry index - crossover hop (%)	93.89 ± 3.92	84.45 ± 4.33	9.44	(7.44, 11.44)	<0.001
Limb symmetry index - timed hop (%)	96.45 ± 2.85	88.34 ± 3.65	8.11	(6.21, 10.01)	<0.001

RTS, return to sport; cm, centimeter; %, percent; CI, confidence interval.

### 3.4 Correlation between gait asymmetry and hop performance

**FIGURE 2:**
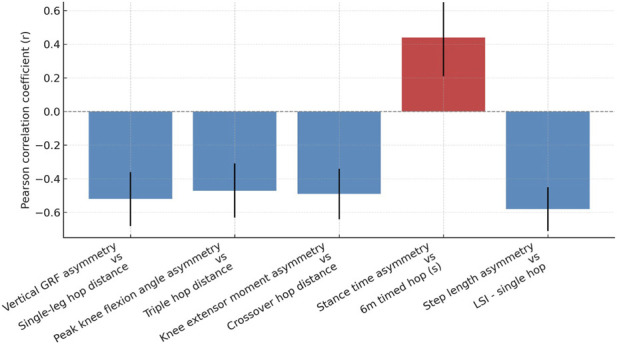
Correlation between gait asymmetry indices and functional hop test performance (r-values with 95% confidence intervals).

Significant correlations were found between greater gait asymmetry and poorer functional hop performance. Vertical ground reaction force asymmetry correlated negatively with single leg hop distance (r = −0.52, 95% CI: −0.68 to −0.32, p < 0.001), and knee extensor moment asymmetry was negatively correlated with crossover hop performance (r = −0.49, 95% CI: −0.64 to −0.28, p < 0.001). Step length asymmetry showed the strongest association with the limb symmetry index in the single hop (r = −0.58, 95% CI: −0.71 to −0.39, p < 0.001), underscoring the biomechanical importance of symmetry for functional outcomes (Table 4; Figure 2).

**TABLE 4:** Correlation between gait asymmetry indices and hop test performance.

Gait asymmetry variable	Hop test performance variable	Pearson r	95% CI	p-value
Vertical GRF asymmetry (%)	Single-leg hop distance (cm)	−0.52	(-0.68, −0.32)	<0.001
Peak knee flexion angle asymmetry (°)	Triple hop distance (cm)	−0.47	(-0.63, −0.25)	<0.001
Knee extensor moment asymmetry (Nm/kg)	Crossover hop distance (cm)	−0.49	(-0.64, −0.28)	<0.001
Stance time asymmetry (%)	6-M timed hop (s)	0.44	(0.21, 0.61)	0.003
Step length asymmetry (cm)	Limb symmetry index - single hop (%)	−0.58	(-0.71, −0.39)	<0.001

GRF, ground reaction force; Nm/kg, N m per kilogram; cm, centimeter; °, degrees; CI, confidence interval; r, Pearson correlation coefficient.

### 3.5 Regression analysis predicting hop performance

**FIGURE 3:**
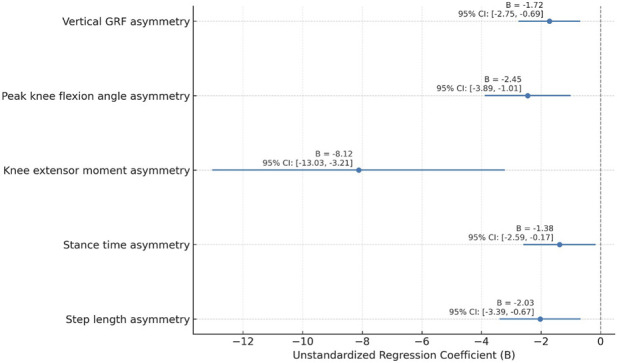
Regression coefficients (B values with 95% confidence intervals) for gait asymmetry metrics predicting functional hop performance.

Multiple linear regression identified several gait asymmetry parameters as significant predictors of functional hop performance. Peak knee flexion angle asymmetry (B = −2.45, 95% CI: −3.89 to −1.01, β = −0.41, p < 0.001), knee extensor moment asymmetry (B = −8.12, 95% CI: −13.03 to −3.21, β = −0.39, p = 0.004), and vertical ground reaction force asymmetry (B = −1.72, 95% CI: −2.75 to −0.69, β = −0.34, p = 0.002) emerged as the strongest predictors. These findings suggest that gait asymmetry metrics can serve as clinically meaningful indicators of impaired functional recovery after ACL reconstruction (Table 5; Figure 3). These findings indicate that gait asymmetry metrics are significantly associated with functional hop performance following ACL reconstruction; however, given the cross-sectional design, these associations should not be interpreted as evidence of causal or longitudinal prediction.

**TABLE 5:** Multiple linear regression analysis predicting functional hop performance from gait asymmetry metrics.

Predictor variable	Unstandardized B	Standard error	95% CI	Standardized beta (β)	p-value
Vertical GRF asymmetry (%)	−1.72	0.52	(-2.75, −0.69)	−0.34	0.002
Peak knee flexion angle asymmetry (°)	−2.45	0.73	(-3.89, −1.01)	−0.41	<0.001
Knee extensor moment asymmetry (Nm/kg)	−8.12	2.45	(-13.03, −3.21)	−0.39	0.004
Stance time asymmetry (%)	−1.38	0.61	(-2.59, −0.17)	−0.28	0.027
Step length asymmetry (cm)	−2.03	0.69	(-3.39, −0.67)	−0.32	0.005

GRF, ground reaction force; Nm/kg, N m per kilogram; cm, centimeter; B, unstandardized regression coefficient; SE, standard error; CI, confidence interval; β, standardized beta coefficient.

## 4 Discussion

This study examined the relationship between gait asymmetry and functional performance after ACL reconstruction, comparing individuals who did and did not return to sport, and assessing associations between asymmetry metrics and hop test outcomes. Individuals who returned to sport demonstrated more symmetrical gait patterns and better hop performance, with greater asymmetry consistently associated with poorer functional outcomes. While several asymmetry variables were statistically associated with functional capacity, these findings reflect cross-sectional associations rather than causal or predictive relationships. Although sex-related differences were explored, no consistent patterns were identified, and the study was not powered to draw definitive conclusions, highlighting the need for larger, stratified investigations.

From a mechanistic perspective, reduced gait asymmetry likely reflects optimal neuromuscular coordination, improved interlimb load distribution, and restored knee joint kinetics during dynamic tasks (Wang et al., 2023). These factors are critical for efficient force generation and attenuation (Maniar et al., 2022; Yu et al., 2026). They directly influence performance in high-demand activities such as hopping and cutting (Mănescu et al., 2025). The observed associations between knee flexion angle and knee extensor moment asymmetry with functional outcomes further suggest that sagittal-plane control of the knee is a key determinant of post-operative performance capacity (Higinbotham, 2025). Clinically, these findings support the incorporation of targeted interventions to restore symmetrical movement patterns, including neuromuscular re-education, eccentric strength training, and movement retraining under task-specific conditions (Kumar Sr et al., 2026). Moreover, integrating objective gait analysis into RTS criteria may enhance current decision-making models, which often rely heavily on time-based or strength-based metrics, by providing a more comprehensive assessment of movement quality and residual biomechanical deficits.

The observed differences in gait asymmetry and functional performance between individuals who returned to sport and those who did not align with prior research, highlighting the biomechanical and neuromuscular disparities that persist following ACL reconstruction (Devana et al., 2022; Hollman et al., 2021). Consistent with the current findings, Ficek et al. reported that athletes who failed to regain pre-injury activity levels often exhibited greater lower-limb asymmetries during dynamic tasks (Ficek et al., 2022). Patients with lower-limb symmetry in kinetic variables, such as ground reaction force and joint moments, were more likely to meet return-to-sport criteria, as demonstrated by Kotsifaki et al. (2022). Reduced gait asymmetry has been closely associated with higher functional readiness and performance, reinforcing the importance of biomechanical restoration in successful rehabilitation (Tajdini et al., 2021). Kinematic symmetry, particularly in knee flexion angles and stance time, has been shown to strongly differentiate high-functioning from low-functioning ACL-reconstructed individuals, further corroborating the present findings (Ptaszyk et al., 2025).

The secondary objective, examining the association between gait asymmetry and functional hop performance, revealed significant correlations and predictive relationships, which are similarly supported by the existing literature. Greater limb asymmetries in joint loading and kinematics have been shown to negatively affect hop test performance, indicating that mechanical deficits are closely linked to impaired function (Cooper, 2023). Kinetic asymmetries have also been associated with reduced performance in single-leg hop tests, suggesting that residual movement asymmetries limit the ability to execute dynamic tasks efficiently (Kotsifaki et al., 2022). Specific gait variables, including vertical ground reaction force and step-length asymmetries, have been shown to predict the limb symmetry index during hop testing (Khoygani and Esmaeili, 2025). These converging findings validate the current study’s regression and correlation results, emphasizing that biomechanical symmetry is not only descriptive of functional status but also predictive of performance outcomes during rehabilitation (Ezzat et al., 2021).

### 4.1 Clinical significance

The present study demonstrated that individuals who returned to sport after ACL reconstruction exhibited significantly lower gait asymmetry and superior functional hop performance compared to those who did not return to sport. Statistically significant between-group differences were observed across key biomechanical variables, including vertical ground reaction force asymmetry (8.45% ± 3.21% vs. 12.34% ± 4.02%, p = 0.001) and peak knee flexion angle asymmetry (3.12 ± 1.10° vs. 5.67 ± 1.45°, p < 0.001). In addition, functional performance measures were significantly better in the RTS group, with higher hop distances and limb symmetry indices (all p < 0.001). Significant associations were also identified between gait asymmetry and functional performance, including correlations between vertical ground reaction force asymmetry and single-leg hop distance (r = −0.52, p < 0.001), and between step length asymmetry and limb symmetry index (r = −0.58, p < 0.001). Furthermore, regression analysis confirmed that peak knee flexion angle asymmetry (p < 0.001) and knee extensor moment asymmetry (p = 0.004) were significant predictors of functional hop performance. From a clinical perspective, these findings indicate that reduced gait asymmetry is strongly associated with improved functional recovery following ACL reconstruction. The results support the integration of objective gait analysis into rehabilitation assessment and return-to-sport decision-making processes. Incorporating symmetry-based biomechanical metrics alongside functional performance tests may enhance the identification of residual deficits, improve monitoring of neuromuscular recovery, and support more informed and objective criteria for progression to high-level physical activity.

### 4.2 Limitations and areas for future research

This study has several limitations. The cross-sectional design precludes causal inference regarding the relationship between gait asymmetry and functional performance. Rehabilitation factors such as intensity, adherence, and adjunct treatments were not controlled, although the groups were comparable in key clinical characteristics. The sample size was sufficient to detect group differences but may not be representative of broader populations. Biomechanical analysis was limited to the sagittal and frontal planes and did not include transverse-plane dynamics or neuromuscular measures. This limits insight into underlying mechanisms. Future studies should include electromyography and longitudinal designs to better understand neuromuscular contributions and recovery trajectories. It also remains unclear whether reduced asymmetry leads to improved function or reflects higher activity levels. Although surgical variability was minimized and graft type did not differ between groups, the study was not designed to assess the independent effects of surgical or sex-related factors. These aspects should be examined in larger, multicenter studies.

## 5 Conclusion

Individuals who returned to sport after ACL reconstruction showed lower gait asymmetry and better hop performance, with asymmetry measures both associated with and predictive of functional outcomes. These findings support the clinical value of gait analysis in rehabilitation and RTS decision-making and underscore the need for multidimensional assessment frameworks. The results also inform individualized rehabilitation strategies and highlight the need for longitudinal research to establish causal relationships and clinically meaningful symmetry thresholds.

## Data Availability

The raw data supporting the conclusions of this article will be made available by the authors, without undue reservation.
